# Polyaniline/Multi Walled Carbon Nanotubes—A Promising Photocatalyst Composite for Reactive Blue 4 Oxidation

**DOI:** 10.3390/polym14183922

**Published:** 2022-09-19

**Authors:** Ashraf H. Farha, Maha A. Tony, Shehab A. Mansour, Ahmed B. El Basaty

**Affiliations:** 1 Department of Physics, College of Science, King Faisal University, Al-Ahsa 31982, Saudi Arabia; 2 Semiconductors Technology Lab, Physics Department, Faculty of Science, Ain Shams University, Cairo 11566, Egypt; 3 Advanced Materials/Solar Energy and Environmental Sustainability (AMSEES) Laboratory, Faculty of Engineering, Menoufia University, Shebin El-Kom 32511, Egypt; 4 Basic Engineering Science Department, Faculty of Engineering, Menoufia University, Shebin El-Kom 32511, Egypt; 5 Basic Science Department, Faculty of Technology & Education, Helwan University, Saray El Qoupa, El Sawah Street, Cairo 11281, Egypt

**Keywords:** polyaniline/multiwall-carbon nanotubes, Reactive Blue 4, oxidation, ultraviolet, photocatalysis, kinetics

## Abstract

For the photocatalytic removal of the Reactive Blue 4 dye from an aqueous stream, new polyaniline/multi walled carbon nanotube nanocomposites (PANI-MWCNTs) were applied as a promising photocatalyst. The PANI-MWCNT nanocomposites were fabricated by aniline oxidation in the presence of MWCNTs using the typical direct oxidation polymerization route. The morphology, the Fourier transform infrared (FTIR) spectra and the UV-Vis absorbance spectra of the fabricated nanocomposites were studied and the attained data confirmed the good interaction between the MWCNTs and PANI matrix. The PANI-MWCNTs nanocomposites were varied according to the wt%, the MWCNTs, which ranged from 0–10 wt% and the corresponding resultant samples are labeled as P-0, P-3, P-5, P-5, P-7 and P-10, respectively. Such composites showed the high potential for the removal of the Reactive Blue 4 dye containing pollutants from wastewater. The starting concentration of the dye pollutants was halved during the first 5 min of UV illumination. The oxidation technique of Reactive Blue 4 over the prepared nanocomposites were processed in a different way and the highest catalytic activity corresponded to P-7. The process reached the complete dye removal in low concentrations of contaminants. The kinetics of the removal followed the pseudo-second order regime which possesses high correlation coefficients with the *k_2_* in the range of 0.0036–0.1115 L.mg^−1^.min^−1^ for the Reactive Blue 4 oxidation. In this regard, the combination of the PANI and MWCNTs showed a superior novel photocatalytic activity in the oxidation of commercial textile dying wastewater, namely Reactive Blue 4. This study is the starting point for future applications on an industrial scale since the successful performances of the PANI-MWCNT on commercial dye oxidation.

## 1. Introduction

Even though the industrialization and urbanization define our modern societies, environmental pollution remains the most important issue that faces such communities. The industrial sector especially, discharges massive amounts of wastewater, and is considered one of the greatest contributors to such types of pollutions [1]. Moreover, among the industries that have a large role in this particular aqueous effluent pollution is the textile industry. Large amounts of wastewater effluents are generated from the textile industry as a result of using massive amounts of water that are applied during the different steps of the fabric dyeing and finishing procedures. Specifically, it is estimated that in order to process one-kilogram of a conventional textile fabric, about 100 L of water are consumed [2,3].

Therefore, the great efforts made by academia and by the industrial sectors are introduced in order to overcome such an environmental deterioration. So, in the textile industry, the processing of the fibers into fabrics or their related products consumes several numbers of synthetic dyes as well as other chemical materials. At the same time, it is reported that approximately more than two hundred thousand tons of dyes per year are consumed in the form of dye effluents because of the inadequate dyeing procedure of the textile industries all over the world [2,4]. Dyes are water soluble organic colored substances that are applied to provide colors to the textiles. Synthetic dyes are widely used in the textile industries [2,5]. The dye color comes from the presence of the so-called chromophore groups in the dye molecular structure which is responsible for the light absorption [6]. The high water solubility of such dyes makes them difficult to be eliminated from the aqueous media that causes aesthetic damage to the water bodies [7,8]. Furthermore, such dyes cause a reduction and/or prevent the light penetration through the water that affects the photosynthetic rates [9,10,11].

Numerous treatment technologies are cited for wastewater remediation of the textile industry. The conventional techniques include biological, membrane filtration, chemical treatment, physical adsorption and critically the chemical coagulation/flocculation technology [8,11]. Although such techniques are resulting in a good dye removal affinity, the formation of derivative environmental contaminations and toxic substances remain their drawbacks [7]. Hence, the search for more reliable, sustainable and eco-friendly technologies is still a hot research topic. The advanced oxidation process (AOP) technologies have been developed to be efficient wastewater oxidation and remediation methodologies [12]. AOPs are identified as powerful oxidation processes that depend on the generation of highly reactive species. The hydroxyl radicals (˙OH) as initiator oxidizing agents, are applied to organic/inorganic contaminants in wastewater and cause their oxidation [13,14]. The AOPs possess the advantage of efficiently destroying organic compounds in non-selective rapid ways through successive oxidation reactions [15]. In addition, (˙OH) radicals are self-eliminated from the water treatment systems due to their very short lifetime, which is approximately a few microseconds [16,17]. Various AOPs are introduced in the literature on the textile wastewater treatments, such as ozonation, Fenton’s reactions, photochemical, photocatalysis (using transition metal ions), electrochemical and ultrasound irradiations [18]. All such AOP methods are based on using a combination of strong oxidizing agents such as O_3_ or H_2_O_2_ with catalysts and/or irradiation such as ultraviolet or visible light [17]. However, the AOP methods still suffer from several challenges such as relatively higher operational costs, high energy consumption, insufficient practice to be extended to full scale applications [19] and the formation of toxic intermediaries that require the final effluent to be turned into a secondary treatment prior to the final disposal. Furthermore, for some certain categories of toxic compounds that resist the hydroxyl radicals, these make the AOPs not very sufficient for their oxidation [17,19,20]. Therefore, due to such disadvantages, an improvement of wastewater treatment using AOP technology is a required development for more economic materials, that are dependent on renewable energy sources. Then, the integration processes can be applied to target the wide range of pollutants in order to extend such systems to commercial and real applications [21].

For instance, the photocatalytic-based AOPs using catalysis that are compromised with augmented polymers in inorganic substances or carbonaceous materials for enhancing the reactive visible light, has attained the interest of scientists [22]. Recently, natural polymer sciences has attracted a greater amount of attention within the research community due to the enormous advantages that are offered by polymer-based materials because of their easy processing, flexibility, recyclability and vastly eco-friendly advantages. Thus, the natural cellulosic fibers from different renewable resources have gained considerable attraction within the research community. Further, the multifunctional nanocomposites based on the conducting and natural polymer nanostructures augmented with other nanoparticles are essential for numerous applications. However, they should be chemically stable with a uniform particle size and possess a high dispersion rate in the aqueous phase [23,24,25,26].

Polyaniline (PANI) is categorized as one of the supreme vital conjugated polymers for its fast charge transfer as well as its fast charge separation capability with a slow charge recombination rate [27,28,29]. Polyaniline, which is signified as one of the conducting polymers in comparison to the conventional polymers, possesses lower band gaps in addition to a fair conductivity. Initially, it received the scientists’ attention due to its unique optical and tunable properties, its environmental stability and its overall friendliness towards the environment since it possesses fused aromatic molecular structures [23,24]. Furthermore, the composites that are based on carbon nanotubes have shown to be efficient visible light photocatalysts for their superior electronic, electrochemical and transport features [30,31].

In this respect, the current work aimed to study the photocatalytic activity of PANI that is loaded with multi-walled carbon nanotubes (MWCNT) on the oxidation of the textile effluent stream. The effect of the concentration of the MWCNTs on the oxidation of the textile effluent stream of the Reactive Blue 4 dye were checked as well as the decomposition process of Reactive Blue 4 was investigated. This investigation is proposed to signify an inspiration to conduct a pathway for improving the photocatalytic performance of MWCNTs. Such results are signified as a driving force to full-scale real industrial applications.

## 2. Experimental Details

### 2.1. Materials

The reagents used in the polymerization process were of the analytical grades with no further purification. The multi-walled carbon nanotubes (MWCNT) with a diameter of (6 to 13 nm) and a length of (2.5 to 20 µm), (Sigma-Aldrich, St. Louis, MO, USA), aniline (Aldrich, Darmstadt, Germany), ammonium persulfate (APS), (BioWORLD, Haryana, India), iso-propyl-alcohol (Merck, Darmstadt, Germany), hydrochloric acid (HCL), (Spectrum, Jalgaon, India), and distilled water are used in the fabrication of the PANI/NC nanocomposites.

### 2.2. Fabrication of the PANI/MWCNT Nanocomposites

The direct oxidation-polymerization method was used in the fabrication of the PANI/MWCNT nanocomposites. The oxidation of aniline in the presence of the MWCNT was carried out in an acidic solution via applying APS as a chemical oxidant. For all of the nanocomposite samples, the acidic medium was maintained. One gram of the total weight from the MWCNT and aniline were used. However, the weight ratio of the MWCNT to aniline was 0:100, 3:97, 5:95, 7:93 and 10:90, respectively, and the obtained nanocomposite samples were labelled as P-0, P-3, P-5, P-7 and P-10, respectively. The sample names and the corresponding weight percentage ratios between the MWCNT to aniline are listed in Table 1. In the typical fabrication method, the desired amounts of the MWCNT and aniline for each sample was added to the earlier prepared acidic aqueous solution (25 mL of distilled water plus 4.35 mL HCL) and subjected to stirring for 20 min. Individually, 2.28 g of APS was added to 25 mL of distilled water and stirred for 20 min. Subsequently, the MWCNT/aniline solution was exposed for sonication (5 min). Thereafter, the APS solution was drop-wise added over about 10 min of reaction time though sonication. The attained solution was subjected to 24 h stirring for in order to complete the polymerization process. Once completed, the nanocomposites were separated by filtration prior to successive washing for three times with distilled water and iso-propyl-alcohol. Then the final product of the PANI/MWCNT nanocomposites was dried in a vacuum overnight at 28 °C.

### 2.3. Characterization Techniques

The morphology of the investigated PANI/MWCNT nanocomposites were examined using a Jeol model JEM-2100 high-resolution transmission electron microscope (HRTEM). The Fourier transform infrared (FTIR) spectra were recorded using a Jasco (Jasco Inc., Tokyo, Japan) FT/IR-4100 spectrophotometer (Jasco Inc., Easton, MD, USA) in the wave number range of 400–4000 cm^−1^. The absorbance spectra of the investigated nanocomposites were measured in the wavelength range of 300–800 nm using a Thermo Scientific Evolution (Thermo Fisher Scientific, Bremen, Germany) 300 UV-VIS spectrophotometer. The measured UV-VIS absorbance was performed for the as-fabricated samples after the dispersion in dimethylformamide (DMF) with a concentration of 1.5 mg/mL.

### 2.4. Oxidation System Set-Up

**Figure 1:**
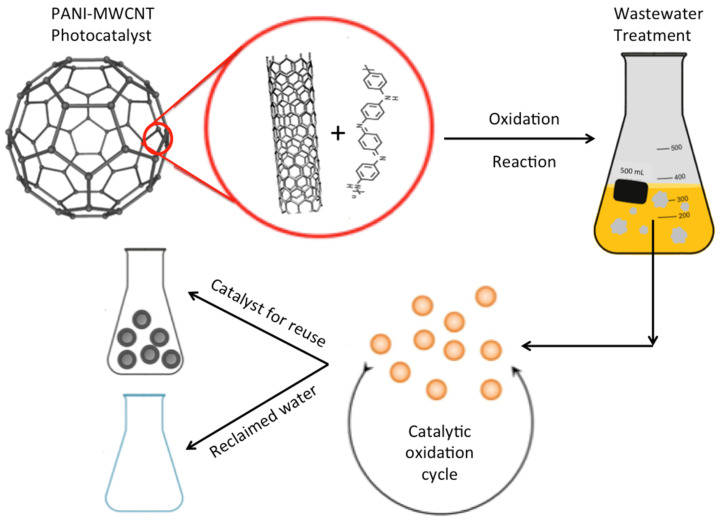
Graphic illustration of the oxidation experimental procedure.

For each oxidation experiment, the100 mL solution (pH 7.0) that contained the Reactive Blue 4 dye at room temperature (28 °C) was poured into a glass container, thereafter the catalyst was added in the range of (10–100 mg/L). Subsequently, the wastewater was subjected to magnetic stirring. An ultraviolet, UV lamp (15 W, 230 V/50 Hz, with 253.7 nm wavelength) was used to emit UV light during the experiments. The lamp was covered with a silica tube jacket to protect the lamp and the UV penetrating into the aqueous media. The sleeved UV lamp was placed inside a glass container containing the aqueous media. Moreover, for the purpose of the comparison with the UV lamp, a white light source Xenon lamp (1000 W high pressure, supplied by Milla, China), was applied. Then, the samples were periodically subjected for analysis prior to filtration via a micro-filter. All of the experiments were run in a triplicate manner. The experimental procedure is graphically illustrated in Figure 1.

### 2.5. Analysis

A UV-vis spectrophotometer (Unico UV-2100 spectrophotometer, Unico, Dayton, NJ, USA) was used to investigate the remaining Reactive Blue 4 concentration at a choice of time intervals at the maximum wavelength of Reactive Blue 4 of 570 nm. Prior to the analysis technique, the polyaniline/multiwall carbon nanotube nanoparticles were detached via a micro-filter.

## 3. Results and Discussion

### 3.1. HR-TEM Micrographs of PANI/MWCNT Nanocomposites

**Figure 2:**
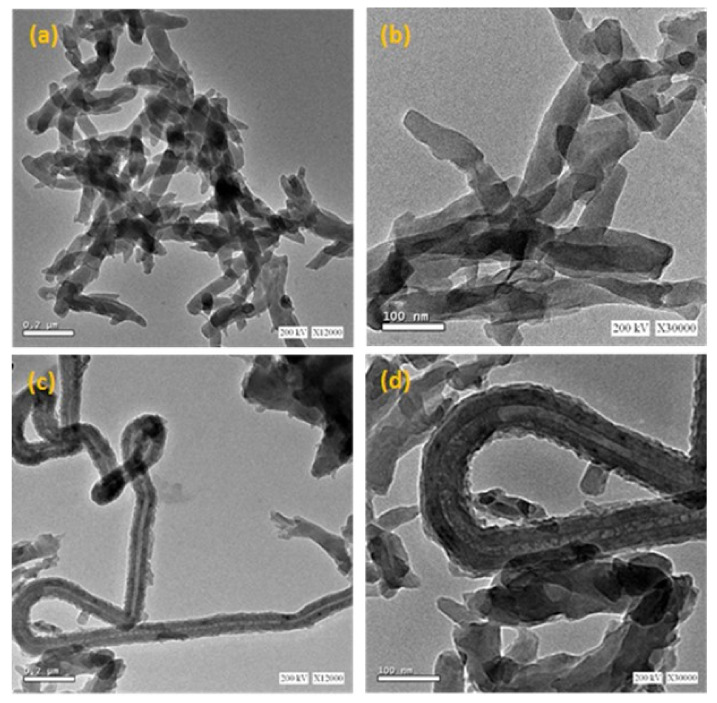
HR-TEM micrographs of (**a**,**b**) for P-0 and (**c**,**d**) for P-5 nanocomposites.

Figure 2 shows the HR-TEM micrographs for the P-0 and P-5 nanocomposites as examples. For the pure PANI sample (P-0), the morphology in Figure 1 and Figure 2a exhibit the formation of ribbon-like nanofiber shapes in an agglomerated form. The obtained nanofibers have a diameter ranging from around 40 to 80 nm, as shown in Figure 2a,b. However, the introduction of the MWCNTs for the P-5 sample, as shown in Figure 2c,d, leads to a variation in its morphology. The obtained micrographs of the P-5 nanocomposite confirmed the achievement of the polymerization over the MWCNTs in the presence of some of the agglomerated regions of the PANI. The presence of coated MWCNTs indicates the good interaction between the PANI and MWCNTs. The obtained arrangement of the PANI nanofiber and the capped MWCNTs leads to an increment in the solid–liquid interfacial region, that helps the oxidation process of the organic pollutant by enhancing the charge transfer rate between the catalysis and pollutant as will be discussed later.

### 3.2. FTIR Spectra Analysis of the PANI/MWCNT Nanocomposites

**Figure 3:**
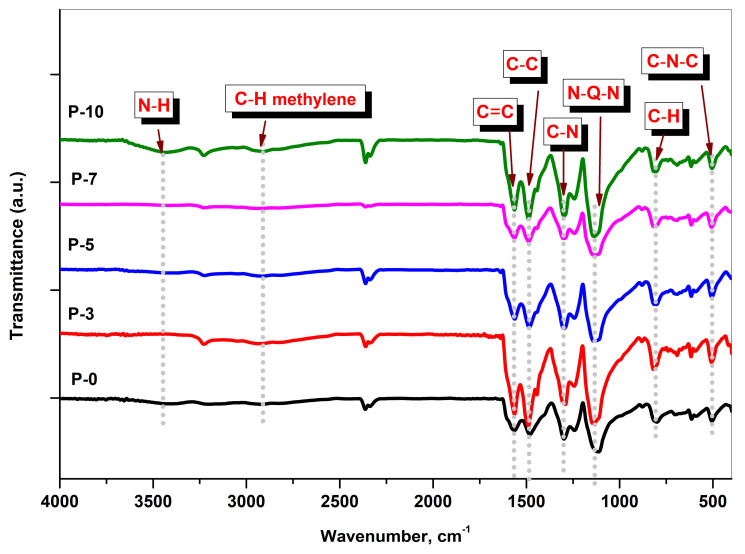
FTIR spectra of the investigated PANI/MWCNT nanocomposites.

Figure 3 shows the FTIR spectra of the PANI/MWCNT nanocomposites. As can be seen from Figure 3, the obtained absorption peaks are in agreement with those reported in the literature for the absorption and vibration modes of the PANI [32]. Such peaks are recorded at 506, 617, 802, 1114, 1298, 1241, 1477, 1564 and 3415 cm^−1^. The absorption bands at 506, 617 and 802 cm^−1^ are due to the amine deformation, the amine in plane deformation and the out-of-plane ring deformation, respectively [33]. The obtained broadening peak at 1114 cm^−1^ assigned to N=Q=N, where Q denotes the quinoid of the PANI. The band at 1298 cm^−1^ ascribed to the C-N stretching mode of the benzenoid rings. The C-N stretching vibrations in the polaronic units manifested by the band at 1241 cm^−1^ [34]. However, both peaks at 1477 and 1564 cm^−1^ are allocated to the C-C stretching mode of the benzenoid rings and the C=C stretching mode of the quinoid rings, respectively [35]. The symmetrical stretching band of the methylene C–H group of the benzene ring is located at 2350 cm^−1^ as indicted in Figure 3. It is worth mentioning that, there are slight shifts for the peaks that are related to the C–C stretching mode of the benzenoid rings to higher values of the wave number with the increase of the MWCNT concentration. This is referring to a strong interaction between the PANI and the MWCNT [36]. The broad band, which appears around 3415 cm^−1^, is assigned to the N–H stretching mode of the benzenoid rings [37].

### 3.3. UV-Vis Spectroscopic Analysis of the PANI/MWCNT Nanocomposites

**Figure 4:**
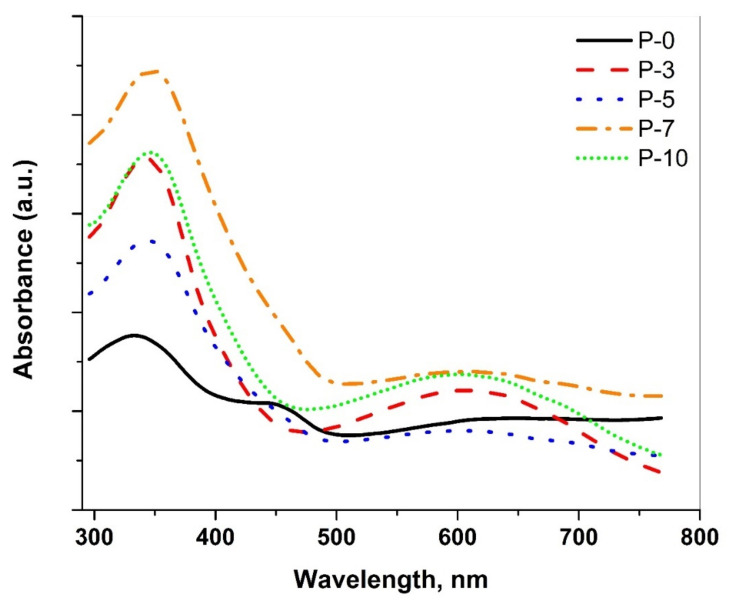
UV-VIS absorption spectra of the investigated PANI/MWCNT nanocomposites.

Figure 4 shows the UV–Vis spectrum of the PANI/MWCNT nanocomposites. As shown in Figure 4, the UV–Vis spectra demonstrate an existence of the π-π* PANI characteristic peak for all samples at ∼333–347 nm due to the excitation of the benzoid segments in the PANI chains [38]. The obtained peaks at ∼600–605 nm are due to the single polaron formation as a result of the excitation of the quinoid segments [39]. The absorption peaks for the PANI/MWCNT nanocomposite samples showed a slight red shift that could be attributed to the interaction between the PANI and the CNT. Moreover, the obtained spectra exhibited an increase in the absorbance as the CNT concentration with the increases in the samples. Such behavior refers to a strong interaction between the CNT and the PANI chain and/or an increase in the combined unsaturated bonds [40]. Another peak ∼448 nm appeared for the pure PANI sample which is assigned to the polaron-π* transition.

**Figure 5:**
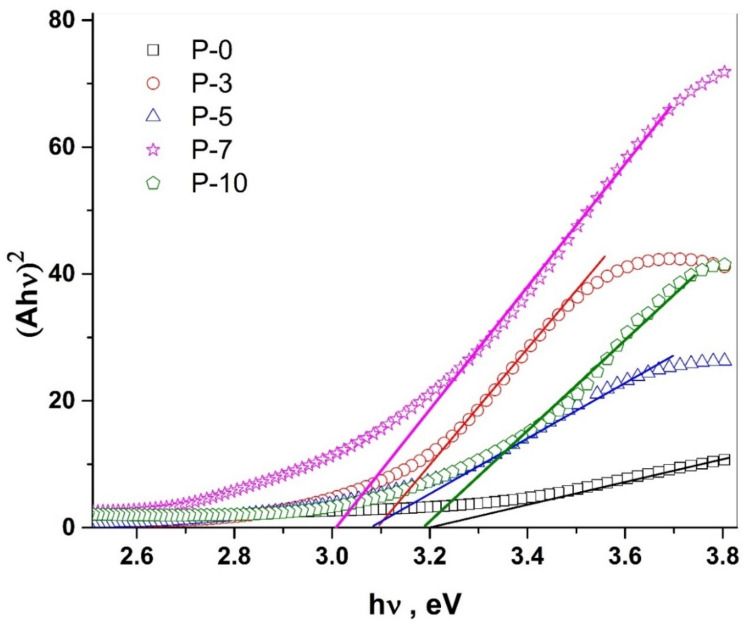
Plot of (Ahv)^2^ versus photon energy (hv) for the PANI/MWCNT nanocomposites.

The UV-vis spectra were used in the estimation of the optical absorption band gap [41]. In this respect, the optical band gap (*E_g_*) of the polymer might be investigated via Equation (1) which gives a relation between the absorbance (*A*) and *E_g_*, which is associated with a particular absorption of photon energy (hυ) [42]. The values of *E_g_* are determined by the extrapolation of the linear portion of ${\left(Ah\nu \right)}^{1/n}$, to zero absorbance.

(1) $${\left(Ah\nu \right)}^{1/n}=h\upsilon -E_{g}$$

where the symbol *n* is associated with the distribution of the density of states and refers to the probable ways of transitions. In the case of the direct allowed transition, *n* is equal to ½. However, *n* is equal to 2 corresponds to the indirect allowed transitions [43]. By applying Equation (1) for the investigated nanocomposite samples, the adequate fits are gained for *n* = ½, as shown in Figure 5. Such a result refers to the existence of a direct allowed band gap transition. The obtained value for *E_g_* is found to be 3.21 eV for the pure PANI sample. For the PANI/MWCNT nanocomposites, *E_g_* showed lower values than the pure PANI sample, P-0, with the non-monotonic trend, as illustrated in Table 1. The energy gap value reached is 3.01 eV for 7% of the MWCNT. The reduction in the band gap with the increasing MWCNT concentration to the PANI host could increase the localized states that directly affect the band gap of the PANI. Indeed, the mobility edge is influenced by the degree of disorder and/or the defects that are presenting in the noncrystalline structure which enables the production of the localized states in the forbidden gap [43].

### 3.4. Photocatalytic Activity of the PANI/MWCNTs on Dye Oxidation

#### 3.4.1. Effect of the Reaction Time and the Initial Reactive Blue 4 Loading

**Figure 6:**
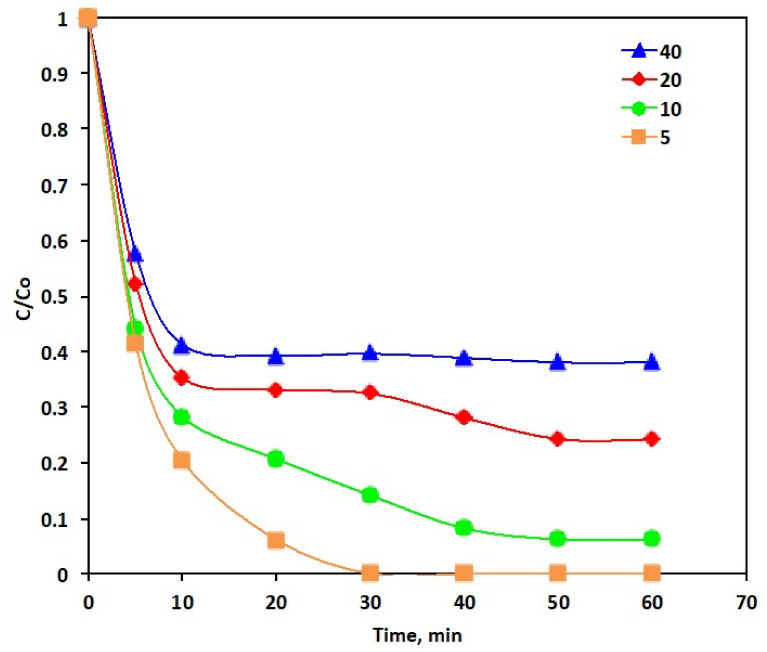
Effect of the initial Reactive Blue 4 loading on the PANI-MWCNTs system (Experimental conditions: PANI-MWCNTs: P-5).

The experimental reaction time that is affecting the reaction kinetics is explored in terms of the Reactive Blue 4 removal efficiency as given by the following equation:

(2) $$Removal\;\left(\%\right)=\frac{\left(C_{0}-C_{t}\right)}{C_{0}}\times 100$$

where: $C_{t}$ is the Reactive Blue 4 concentration at time *t*; *C*_0_: is the initial Reactive Blue 4 concentration. In order to investigate the optimal reaction time and its impact on the oxidation procedure, experimental tests were conducted for the reaction times ranged from 5 to 60 min, while the initial concentration of the catalyst of the PANI/MWCNTs was kept constant (P-5) at 100 mg/L. The glance at Figure 6 elucidates the effect of the reaction time on the profile of various Reactive Blue 4 concentrations. By fixing the data from Figure 6, it displays that the oxidation efficiency of 5 ppm of the Reactive Blue 4 is high in the initial 5 min of illumination time where it reaches to 58%. Next, the high oxidation rate decreases and a cumulative removal efficiency of 100% within 30 min is attained. According to the previous data that were cited in the literature, the Reactive Blue 4 molecules consist of aromatic rings. The Hydroxyl radicals generated from the reaction between P-5 nanoparticles might attack those aromatic rings and open them, generating intermediates of the oxidation reaction and eventually mineralizing them to non-toxic substances, i.e., CO_2_ and H_2_O. However, as the reaction proceeds, the hydroxyl free radical (˙OH) loading is reduced. Furthermore, the different radicals are created that hinder the reaction rate instead of improving the Reactive Blue 4 oxidation.

Meanwhile, the same trend regarding the fast oxidizing rate is investigated at the first time-profile stage that is quite similar for all Reactive Blue 4 loadings. Figure 6 shows that the oxidation rate diminished with the rising Reactive Blue 4 concentration. The oxidation efficiencies are 100, 93, 76 and 62% for 5, 10, 20 and 40 ppm for the Reactive Blue 4 loadings in the aqueous effluent, respectively. This could be attributed to the higher Reactive Blue 4 dye concentrations. The amount of generated ˙OH species is inadequate for the complete Reactive Blue 4 elimination. Moreover, the excess dye in the solution could act as a shadow that prevents the light from penetrating into the aqueous solution in the photo-reactor. Hence, the light photons’ adsorption is reduced as the dye load increases. Thus, the overall radicals’ produced is reduced and the reaction yield has also decreased. Thus, Reactive Blue 4 plays a significant role in the PANI/MWCNTs photocatalytic activity since its load increase decreases the catalytic activity.

This investigation into the enhancement of the photo-oxidation activity with the decreasing the initial pollutant load has also previously been cited by Cetinkaya et al. [44], whereas Fenton’s treatment for the oxidizing textile wastewater effluent was applied. Furthermore, the 30 min of reaction time is enough to attain such removals for the Reactive Blue 4 oxidation, afterwards, a plateau is achieved in all tests. Nonetheless, with extending oxidation reaction irradiance time, the Reactive Blue 4 dye oxidation decelerated and its oxidizing efficacy turn out to be alleviated.

#### 3.4.2. Effect of the PANI/MWCNTs Concentration

As is identified by [45], the strong ˙OH radical oxidative species is responsible for the oxidation reaction. Previous findings by Wang et al. and Arimi et al. [46,47] reported that the variation of the catalyst concentration has a substantial effect on the hydroxyl free radical generation which then affects the overall oxidation efficiency yield. Thus, such a PANI-CNTs dose was examined in this study.

**Figure 7:**
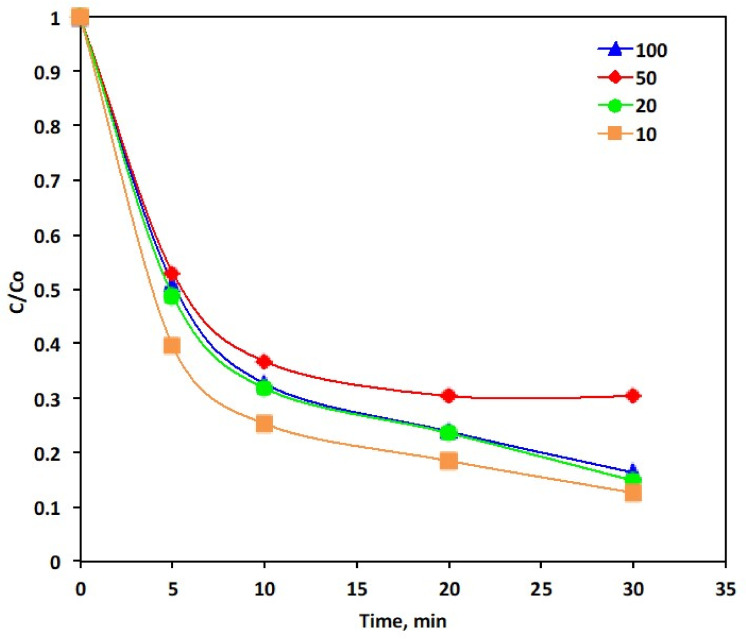
Effect of the PANI-MWCNTs concentration of the Reactive Blue 4 oxidation (Experimental conditions: Reactive Blue 4: 10 ppm; PANI-MWCNTs: P-5).

Figure 7 displays the dye oxidation through their concentration removal at different (10, 20, 50 and 100 mg/L) PANI-MWCNTs dosages in the presence of P-5 of the PANI-MWCNTs and the dye concentration of 10 ppm. The concentration of the dye removal is increasing from 69 to 87% as the PANI-MWCNTs dosage changes and the highest removal corresponds to 10 mg/L.

When the mixture of the PANI-MWCNTs and the dye was placed under ultraviolet radiation, an electron excited (e^−^) from the valence band (VB) to the conduction band (CB) generated a hole in the VB instantaneously. The superoxide radical anion is created by the inhibition of the molecular oxygen present on the photocatalyst’s surface. Then, a further combination of the superoxide radical anion with hydrogen ions (H^+^) occurs in the aqueous medium and the HO_2_˙ radical is generated. Further attacks occur from the trapped electrons into the HO_2_˙ radicals to form OH radicals that are responsible for the photo oxidation reaction [45].

Since the yield of ˙OH is expected to attain an optimal catalyst dose, which leads to an enhancement in the reaction efficiency and the oxidation yield of the dye molecules is increased. Moreover, the hydroxyl (˙OH) radicals are trapped by the PANI-MWCNTs catalyst in excess. Furthermore, the increase in the oxidation rate with the PANI-MWCNTs catalyst load could be due to the large surface area and the associated available sites in order to conduct the oxidation reaction. Furthermore, the possible photocatalytic mechanism could be due to the electron/hole separation that was produced through the synergistic outcome between the PANI and the MWCNTs [22].

#### 3.4.3. Effect of the Various Carbon Concentrations on the PANI/MWCNTs Catalyst

For a photocatalyst, light absorption is the most crucial influencing parameter [48]. Since band-edge potential levels of two semiconductors possess a vital position in defining the photo-excited charge carriers in a coupled hetero-structure. Different combinations of the two catalysts are checked to investigate their band positions effect on the catalytic oxidation. In order to identify the effect of the photo oxidation catalytic activity of the various PANI–MWCNT composites, different compositions of samples are compared and checked for their dye removal efficiency. The samples signified as: P-0, P-1, P-3, P-5, P-7 and P-10 using 50 mg/L of the catalyst dose.

**Figure 8:**
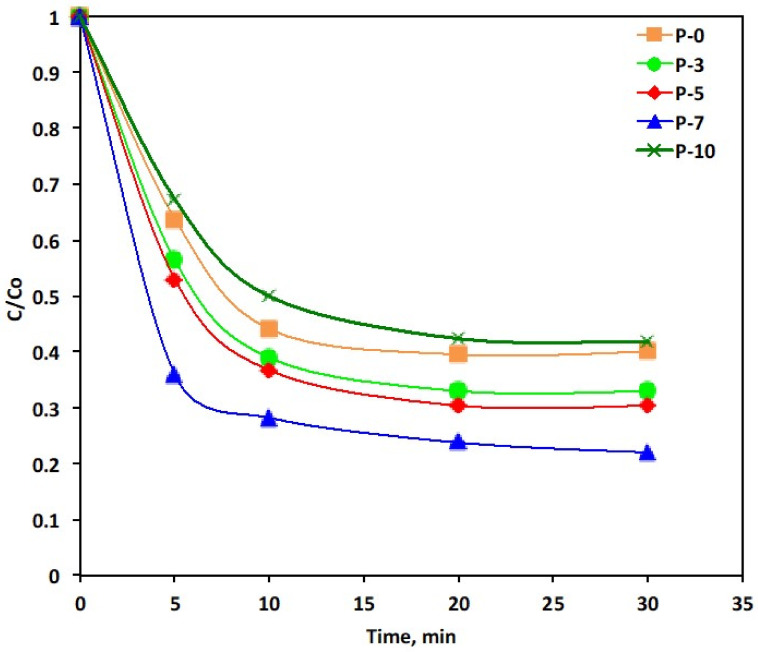
Effect of the different wt% composites and PANI-MWCNTs systems on the Reactive Blue 4 oxidation (Experimental conditions: Reactive Blue 4 = 10 ppm; PANI-MWCNTs: P-5 = 10 mg/L).

Figure 8 illustrates that all of the studied PANI/MWCNTs composites have an enhanced photocatalytic activity expectation of the P-10 sample over the PANI. The maximal removal efficiency belongs to P-7, which reaches 78% of the dye removal efficiency from the aqueous stream. This result is confirmed by the increase of the localized states in the energy gap as discussed in the UV–vis absorbance results. Such an increase in the localized states in the energy gap enables the charge transfer between the catalysis and the dye that is facilitated by the improvement in the photocatalytic activity. However, the excess of the MWCNTs decreases the catalytic reaction of the PANI, which is attributed by increase of *E_g_*, as illustrated in Table 1. The phenomenon attained is in accordance with the previous data that was reported by Lu et al. [49], whose main results are in treating phenolic compounds. Furthermore, the improved activity of the nanocomposites may be associated with the presence of the MWCNT that increases the available surface for pollutant adsorption besides the oxidation reaction. Introducing the functional group such as –COOH into the MWCNTs, changes its surface for being specific to a reaction that is leading to a selective treatment system [50]. Additionally, the addition of the MWCNTs motivates both the C-doping and photosensitization [51]. The suggestive mechanism includes the following steps [22,52,53]:

(3) $$PANI+hv\to e_{CB}^{-}+h_{vB}^{+}$$

(4) $$MWCNTs+hv\to e_{CB}^{-}+h_{vB}^{+}$$

(5) $$Dye\to Dye^{*}$$

(6) $$Dye^{*}+Dye^{+}\to (e_{CB}^{-})MWCNTs$$

(7) $$Dye^{*}+\left(h_{vB}^{+}\right)MWCNTs/PANI\to Dye^{+}$$

(8) $$(e_{CB}^{-})MWCNTs+O_{2}\to O_{2}^{˙-}$$

(9) $$O{˙}_{2}^{-}+e_{CB}^{-}+2H^{+}\to ˙OH+OH^{-}$$

(10) $$˙OH+O{˙}_{2}^{-}+Dye^{+}\to harmless\;end\;products$$

(11) $$Dye+\left(h_{vB}^{+}\right)MWCNTs/PANI\to hermless\;end\;products$$

As mentioned above, when the mixture of the PANI and MWCNTs are induced by ultraviolet radiation, an electron in the conduction band (CB) is created and also a hole in the valance band (VB) is produced, according to Equations (3) and (4). Further, the dye pollutant in the aqueous media is converted into a positive charge, which reacts with the activated MWCNT (Equations (5)–(7)). As a result of the activation, the activated CB in the MWCNTs reacts with the trapped oxygen and hence the oxygen anion is generated ($O_{2}^{˙-}$) (Equations (8) and (9)). Moreover, the hydrogen ion in the solution is combined with the oxygen radical and electron in the conduction band to generate both radicals (˙OH and ${\mathrm{OH}}^{-})$ as given by Equation (10). Such hydroxyl radicals (˙OH) are oxidizing the dye species in the aqueous solution (Equation (11)) into harmless end products which are carbon dioxide and water. Furthermore, the hole in the valance band in the MWCNTs/PANI also reacts with the dye in the aqueous solution and produces harmless end products (Equation (11)).

#### 3.4.4. Comparison of the Different Light Sources

**Figure 9:**
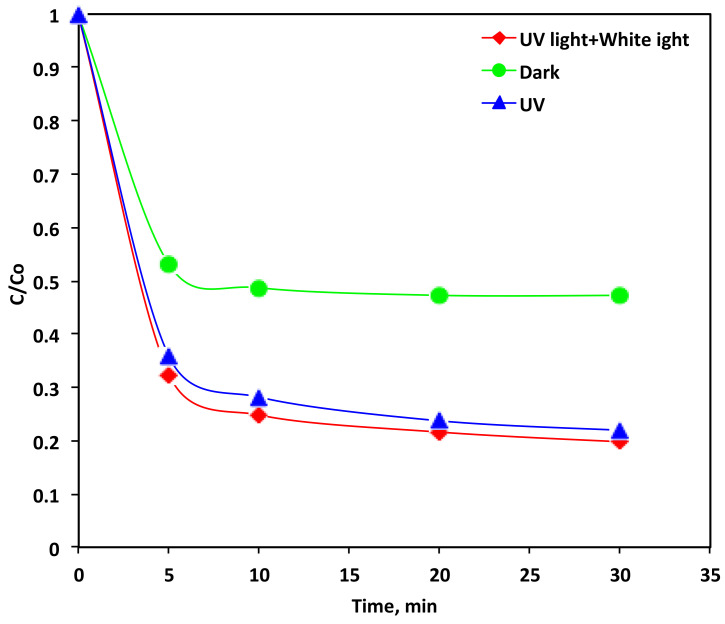
Effect of the different light sources on the Reactive Blue 4 oxidation by the PANI-MWCNTs system (Experimental conditions: Reactive Blue 4 = 10 ppm; PANI-MWCNTs: P-5 = 10 mg/L).

Photocatalytic oxidation technology is linked to the ultraviolet light, which is considered the key parameter in the photocatalytic reactions. Light might be from the natural UV source, which is a naturally abundant and cheap source (sun) or it may be an artificial source of ultraviolet illumination. UV radiation is signified as the key in photocatalytic reactions. In laboratory assays, white light that simulates the natural solar light was applied and compared with ultraviolet light. For the purpose of comparing and investigating both the white light and UV light effects, the dark experiment is conducted to reveal their effectiveness on wastewater treatment. Therefore, such laboratory-scale examinations are helpful for evaluating in pilot/full-scale the management technology for real treatments using sun as a solar energy. Figure 9 displays the Reactive Blue 4 oxidation by using three irradiation cases (a) UV light, (b) white light + UV light and (c) the dark system. The results reveal that the augmentation of the two light sources, namely the white light and ultraviolet sources could improve the hydroxyl radical production. Hence, the oxidation reaction rate was enhanced from 53% for the dark oxidation test to 78% for the UV illumination reaction and that increased to 80% for the combined white light + UV light system. It is recommended that the light wavelengths emitted through the combined light sources are better for the photo oxidation of Reactive Blue 4 in comparison with the solo UV wavelength emitted by the ultraviolet lamp [54]. Thus, it could be suggested that when the UV and white lights stimulate photoreceptors, extra (OH) radicals are generated. It is noteworthy to mention that the dark oxidation reaction also reveals a relatively high dye elimination that reaches to 53%. This confirms that the reaction is a simultaneous oxidation and adsorption reaction. Although, in the presence of the UV or white light, more radicals are enhanced and thus the reaction yield is further increased to 78% and 80% for the UV illumination and combined white and UV light system, respectively. This confirms the photons captured due to the presence of how light plays the role in enhancing the electrons in the VB and the holes in CB in the PANI/MWCNTs for successive reactions, which includes the generation of OH radicals.

A comparison of the pollutant oxidation using the modified PANI-MWCNTs as catalyst with other treatment tools is tabulated in Table 2. The oxidation efficiency of the various PANI based systems and their numerous composites were evaluated and compared with the PANI-MWCNTs from the current investigation. Additionally, the results from the current study showed a superior efficiency than other techniques. The present work also showed a lower oxidation irradiance time in comparison with other photocatalysts. The PANI-MWCNT oxidation showed that an efficient treatment reached 100% for the color removal. Although some other technologies that were completed earlier, displayed their potential through studies using high oxidation for the removal of contaminants from wastewater. They show some pitfalls, such as the requirement of a higher irradiance time that makes the process costly. Such problems are not associated with the current treatment technology suggested for the Reactive Blue 4 dye effluent oxidation. However, it is also worth mentioning that various experimental circumstances such as pH, temperature and the type of UV lamp used may affect the reaction rate. Furthermore, the different types of contaminants originate from various oxidation tendencies, which also affect the reaction rate. However, the comparison is conducted by comparing the optimized conditions in each case.

The significant feature of this investigation is the MWCNTs/PANI composite re-usability. In this regard, the catalyst re-generation is conducted through collection after use. The collected catalyst is filtered prior to it being subjected to three sequential washings with distilled water and then dried in an oven (110 °C, 1 h). The successive cycles after the catalyst use in the treatment is checked for its reactivity by inspecting the dye removal. The catalyst showed good reusability was reached after its sixth cycle and the removal efficiency reached 70% compared with 100% prior to the first use of the catalyst. This could be illustrated by some organic intermediates that covered those active centers and prevented them from producing hydroxyl radicals or that attacked the dye molecules [55]. Thus, the overall reaction yield has decreased. However, it is worth mentioning that such results confirm the catalysts’ sustainability since a reasonable treatment is achieved, even for the successive use of the catalyst.

#### 3.4.5. Kinetic Determination of the PANI/MWCNTs Oxidation

From the practical application perspective, and for achieving higher oxidation levels, analyzing the PANI-MWCNTs oxidation kinetics for the oxidation system is a very important theme. Attaining the fitting kinetics results, the optimal operational sets, the economic cost, the system control and a scale-up photo-reactor design might be suggested [66]. Thus, the photo-catalytic oxidation of the Reactive Blue 4 aqueous effluent was investigated through the kinetic examination with varying the amounts of the initial Reactive Blue 4 dye concentrations. The association between the various initial loading and the time of the pseudo first- and second-order reaction rates, respectively, are investigated and the results are displayed in Table 3. Table 3 tabulated the pseudo-first- and second-order rate constants (*k*_1_ and *k*_2_), and the reaction half-life time (*t*_1/2_) for the treatment system at various initial loadings.

It is verified in Table 3 that, for the two kinetic models, the rate constants are elevated with decreasing the initial Reactive Blue 4 concentration, but the *t*_1/2_, decreased. This could be related to the occurrence of the dye molecules in excess in the aqueous stream in the case of a higher dye concentration. The result is a decrease in the overall hydroxyl radicals generated, that means they diminish the overall reaction yield [67].

Based on the values of regression coefficients (*R^2^*) listed in Table 3, it displays the *R^2^* values for the pseudo second-order reaction rate which ranges from 0.82 to 0.99 for the data collected using different concentrations from 5 to 40 ppm. However, the corresponding *R^2^* for the pseudo first-order reaction values are lower. This confirms the influence of the initial concentration of the catalytic oxidation Reactive Blue 4 in an aqueous stream via the PANI-MWCNTs proceeds in accordance with the pseudo second-order kinetic model. This investigation is in agreement with those previously cited in the literature [68], in treating azo dye RY84 with a catalytic oxidation.

## 4. Conclusions

In summary, a facile synthesis of the PANI/MWCNTs composite has been prepared for eliminating dye in an aqueous effluent. The PANI loaded by MWCNTs in different weights % is prepared and the results show that up to 10% were successfully fabricated with a good interaction between them. The UV-Vis absorbance of the PANI/MWCNT test revealed a reduction in the energy gap with increasing the MWCNT in the composite, which is due to the presence of the localized states through the band gap of the PANI. The application of the PANI/MWCNTs showed a high oxidation efficiency through the elimination of Reactive Blue 4 from the aqueous stream that could be reached to a complete (100%) of the dye removal within 30 min of irradiance time. Comparing the dark test with the UV illumination and white light revealed that the dark test could reach 53% removal, however the highest removal efficiency is associated with the UV illumination which confirms the role of both the photocatalytic reaction and the adsorption test. P-7 that revealed the best MWCNTs added wt. (7%) to the PANI particles. The kinetic modeling confirms the reaction follows the second-order kinetic model. Moreover, the catalysts’ reusability verifies the sustainability of the catalyst, which confirms the suitability of the catalyst for real-life industrial applications.

## Figures and Tables

**Table 1:** Weight ratio of the MWCNT to aniline and the calculated optical energy gap for the investigated samples.

Sample Name	Weight Ratio of MWCNT to Aniline	*E_g_* (eV)
P-0	0:100	3.21
P-3	3:97	3.095
P-5	5:95	3.09
P-7	7:93	3.01
P-10	10:90	3.10

**Table 2:** Comparison of the oxidation boundaries using different photocatalysts under dark, visible and ultraviolet (UV) light irradiation for the removal of various pollutants.

Name of the Photocatalyst	Name of the Pollutant	Irradiation Source	Irradiation Time	Oxidation Efficiency (%)	Ref.
PANI-MWCNTs	Reactive Blue 4	UV light	30 min	100%	Current work
PANI-MWCNTs	Reactive Blue 4	Dark oxidation	30 min	53%	Current work
PANI-TiO_2_	Methyl orange	Visible light	120 min	49%	[56]
PANI modified-TiO_2_	Methyl orange	UV light	125 min	81%	[57]
n-CuO nanoparticles	Methomyl pesticide	UV light	15 min	99%	[53]
Nanostructured TiO_2_/ZnO heterojunctions	Methyl orange	UV light	80 min	97%	[58]
TiO_2_/graphene	Methyl orange	UV light	60 min	85%	[59]
TiO_2_ nanoparticles	Methyl orange	Visible light	120 min	76%	[29]
p-CuO/n-ZnO	Methyl orange	Visible light	150 min	81%	[60]
Fe_2_O_3_ nanocrystals/H_2_O_2_	Bismarck Dye	UV light	10 min	78%	[61]
Iron-coated sand catalyst	Organics	Dark oxidation	30 min	70%	[62]
PANI/Al-ZnO	Methyl orange	Visible light	150 min	92%	[63]
Cu_2_O/ZnO	Red dye	Solar light	180 min	93%	[57]
PANI/Al-ZnO	Methyl Orange	Visible light	150 min	92%	[63]
Fe_2_O_3_ nanocrystals	Bismarck dye	Solar light	60 min	59%	[52]
Fe_2_O_3_/H_2_O_2_	Procion Blue	UV light	60 min	76%	[64]
PANI/Co-doped ZnO nanocomposites	Procion Blue	UV light	425 min	84%	[65]

**Table 3:** Kinetic parameters of the Reactive Blue 4 wastewater oxidation via the PANI-MWCNTs under different concentrations *.

Concentration, ppm	First-Order Reaction Kinetics Model			Second-Order Reaction Kinetics Model			Removal, %
	$C_{t}=C_{0}-e^{k_{1}t}$			$\left(\frac{1}{C_{t}}\right)=\left(\frac{1}{C_{0}}\right)-k_{2}t$			
	*k*_1_, min^−1^	*R* ^2^	*t*_1/2_, min	*k*_2_, L mg^−1^ min^−1^	*R* ^2^	*t*_1/2_, min	
5	0.1126	0.96	6.15	0.1115	0.93	44.84	100
10	0.0554	0.90	12.50	0.026	0.98	384.61	84
20	0.0319	0.64	21.72	0.0049	0.82	4081.63	67
40	0.0262	0.61	26.45	0.0036	0.99	11,111.11	60

* *C_0_* and *C_t_*: initial and at time *t*; *t*: time (min); *k*_0_, *k*_1_, *k*_2_: kinetic rate constants of zero-, first- and second-reaction kinetic models.

## Data Availability

All data generated or analysed during this study are included in this published article.
